# Clinical significance of the cachexia index in patients with small cell lung cancer

**DOI:** 10.1186/s12885-021-08300-x

**Published:** 2021-05-17

**Authors:** Se-Il Go, Mi Jung Park, Gyeong-Won Lee

**Affiliations:** 1 Division of Hematology-Oncology, Department of Internal Medicine, Institute of Health Science, Gyeongsang National University Changwon Hospital, Gyeongsang National University College of Medicine, Changwon, Republic of Korea; 2 Department of Radiology, Gyeongsang National University Hospital, Gyeongsang National University College of Medicine, Jinju, Republic of Korea; 3 Division of Hematology-Oncology, Department of Internal Medicine, Institute of Health Science, Gyeongsang National University Hospital, Gyeongsang National University College of Medicine, 79 Gangnam-ro, Jinju, 52727 Republic of Korea

**Keywords:** Small cell lung carcinoma, Cachexia, Sarcopenia, Serum albumin, Biomarker

## Abstract

**Background:**

Cancer cachexia worsens the treatment outcomes of patients with small-cell lung cancer (SCLC). However, no reliable biomarker of cancer cachexia is yet known.

**Methods:**

We retrospectively evaluated male SCLC patients who received induction chemotherapy or concurrent chemoradiotherapy. The cachexia index (CXI) was calculated as skeletal muscle index × serum albumin level (g/dL)/neutrophil-to-lymphocyte ratio. The CXI cutoff according to tumor stage was determined based on a time-dependent receiver operating characteristic curve, and all patients were divided into low- and high-CXI groups.

**Results:**

Of 267 patients, 83 and 24 patients with limited-stage disease (LD) and 123 and 37 patients with extensive-stage disease (ED) were assigned to the high- and low-CXI groups, respectively. Only one of 24 patients (4.2%) with LD in the low-CXI group achieved a complete response (CR), whereas 30 of 83 patients (36.1%) with LD in the high-CXI group achieved CRs (*p* = 0.004). More low-CXI patients required early discontinuation of treatment because of treatment-related toxicity compared to the high-CXI patients (37.5% vs. 16.9%, respectively, *p* = 0.030, for LD patients; 27.0% vs. 11.4%, respectively, *p* = 0.019, for ED patients). The median progression-free survival (PFS) and overall survival (OS) were significantly shorter in the low-CXI group than the high-CXI group (6.3 vs. 11.1 months and 7.5 vs. 20.6 months, respectively, both *p* <  0.001 for LD patients; 2.9 vs. 6.3 months and 5.8 vs. 12.8 months, respectively, both *p* <  0.001, for ED patients). On multivariate analysis, low-CXI status was an independent poor prognostic factor for both PFS and OS regardless of the tumor stage.

**Conclusion:**

A low CXI was associated with treatment intolerance, poor treatment response rate, and poor prognosis in SCLC.

**Supplementary Information:**

The online version contains supplementary material available at 10.1186/s12885-021-08300-x.

## Background

Small cell lung cancer (SCLC) is a highly aggressive disease characterized by rapid tumor growth, early widespread dissemination, and a high probability of relapse [1, 2]. Although the response duration is short, etoposide or irinotecan plus platinum-based chemotherapy with or without concurrent radiotherapy is associated with a high response rate and prolongs survival [3–5]. The addition of immunotherapy to chemotherapy further improved the treatment outcomes of patients with extensive-stage disease (ED) [6, 7]. However, elderly patients or those with poor performance status (PS) experience more treatment-related toxicity and tend to be unable to undergo optimal treatment [8–10]. As the prognosis of SCLC patients who fail to complete treatment is extremely poor, those who cannot tolerate induction chemotherapy should be identified and treated with intensive supportive care.

Cancer cachexia (an ongoing loss of skeletal muscle mass that cannot be fully reversed by conventional nutritional support [11]) is associated with more treatment-related toxicity, a reduced quality of life, and poor prognosis [12–14]. In SCLC patients, weight loss is associated with a poor treatment response, decreased quality of life, and short survival [15–17]. Several biomarkers of cachexia, such as sarcopenia, cachexia score, and nutritional indices, have been suggested to be prognostic in SCLC patients [18–22]. The cachexia index (CXI) is a novel measure of cachexia in patients with advanced non-small cell lung cancer and non-Hodgkin’s lymphoma [23, 24]. The CXI considers the skeletal muscle index (SMI), serum albumin level, and neutrophil-to-lymphocyte ratio (NLR), and may thus comprehensively reflect cachectic status. Currently, any role for the CXI in SCLC remains unclear. Therefore, we investigated whether the CXI reflected the prognosis and treatment outcomes of SCLC patients.

## Methods

### Patients

From July 2006 to June 2020, all consecutive male SCLC patients receiving etoposide or irinotecan plus platinum combination chemotherapy as first-line treatment (with or without radiotherapy) in a single institution were retrospectively reviewed. As the CXI cutoff likely differs according to sex because muscle mass varies by sex, female patients were excluded as the sample size was too small to determine their cutoff. Patients with another type of cancer and/or a serious active infection were excluded. Those for whom serum albumin levels and complete blood counts measured within 7 days before the first cycle of chemotherapy as well as baseline chest computed tomography (CT) scans were unavailable were excluded. The study was approved by the Institutional Review Board of Gyeongsang National University Hospital.

### Assessments

Clinical, laboratory, and radiological data were extracted from electronic medical records. The CXI was calculated as SMI × serum albumin level (g/dL)/NLR [23]. We used the pectoralis major and minor muscles to measure the SMI based on a previously described method [19]. Briefly, the region of interest (ROI) was drawn freehand at the outermost border of the pectoralis muscles at the T4 level, and its area, ranging from − 29 to 100 HU, was calculated via CT histogram analysis (“X section” analysis tool, Advantage Window 4.4; GE Healthcare, Milwaukee, WI, USA). The cross-sectional areas of the bilateral pectoralis muscles were separately calculated based on CT histograms. The average area was normalized to height (m^2^); the SMI thus had the unit cm^2^/m^2^. Underweight was defined as a body mass index (BMI) <  18.5 kg/m^2^ (the Asian criterion). The response to anticancer therapy was assessed using the Response Evaluation Criteria in Solid Tumors (RECIST) ver. 1.1 [25]. The objective response rate (ORR) was calculated as the proportion of patients who achieved complete response (CR) or partial response (PR). All eligible patients were included in the analyses of treatment response, progression-free survival (PFS), and overall survival (OS) regardless of whether their treatment response was evaluated radiologically. Therapy-related adverse events were assessed using the National Cancer Institute Common Toxicity Criteria for Adverse Events ver. 4.0. Treatment-related mortality (TRM) was defined as death from any cause other than cancer progression before 30 days after the last cycle of first-line chemotherapy. Early discontinuation of treatment was noted when first-line chemotherapy ceased because of treatment-related toxicity, regardless of the response to treatment.

### Statistical analysis

The CXI cutoff was determined by maximizing the Youden index (the sum of sensitivity and specificity) for predicting 18- and 10-month survival in limited-stage disease (LD) and ED, respectively, using a time-dependent receiver operating characteristic (ROC) curve [26]. Each survival time point for determining the cutoff was decided by considering historical data [3, 5, 27, 28]. With reference to the cutoffs, patients were divided into low- and high-CXI groups. Correlations between dichotomous and continuous or categorical variables were explored using the Mann-Whitney U-test and the chi-squared test as appropriate. OS was defined as the time from the first day of treatment to death or the last follow-up. PFS was calculated as the time from the first day of treatment to progression, death, or the last follow-up. The Kaplan-Meier method and the log-rank test were used to estimate survival data. A Cox’s regression model was employed for multivariate analysis. All variables with *p*-values < 0.10 on univariate analyses were included in the multivariate regression model. A two-sided *p*-value < 0.05 was considered to indicate statistical significance. All statistical analyses were performed using R ver. 3.6.2 (R Foundation for Statistical Computing, Vienna, Austria) and STATA ver. 16.1 (College Station, TX, USA).

## Results

### Patient characteristics

The mean (± standard deviation) and median (interquartile range) CXI values were 11.09 (± 6.17) and 10.08 (6.30–14.24) for LD patients and 8.18 (± 5.92) and 6.77 (3.94–11.06) for ED patients, respectively (*p* <  0.001). The area under the curve (AUC) (calculated using time-dependent ROC data) was 0.632 [95% confidence interval (CI) 0.521–0.744] for LD and 0.665 (95% CI 0.577–0.753) for ED (Supplementary Fig. 1). The CXI cutoffs were 5.82 (sensitivity 35.1% and specificity 92.5%) for LD and 3.83 (sensitivity 43.5% and specificity 90.9%) for ED. In total, 83 and 24 patients with LD and 123 and 37 patients with ED were assigned to the high- and low-CXI groups, respectively.

Comparison of patient baseline characteristics between the high- and low-CXI groups are presented in Table 1. Among the LD patients, the median age was higher in the low-CXI group than in the high-CXI group (70.5 vs. 66 years, respectively, *p* = 0.033), whereas there was no difference between the two groups for ED patients. The proportion of patients classified as Eastern Cooperative Oncology Group (ECOG) PS 2–3 was higher in the low-CXI group than in the high-CXI group regardless of stage (33.3% vs. 10.8%, respectively, *p* = 0.008, for LD patients; 40.5% vs. 20.3%, respectively, *p* = 0.013, for ED patients). Among ED patients, the low-CXI group received prophylactic cranial irradiation less frequently (16.2% vs. 40.7%, respectively, *p* = 0.006) and had a lower median BMI (21.0 vs. 22.6 kg/m^2^, respectively, *p* = 0.007) compared to the high-CXI group.

**Table 1:** Comparison of baseline characteristics between the high- and low-CXI groups

Characteristic	LD			ED		
	High-CXI group (*n* = 83)	Low-CXI group (*n* = 24)	*p*	High-CXI group (*n* = 123)	Low-CXI group (*n* = 37)	*p*
Age			0.067			0.214
< 70 years	52 (62.7%)	10 (41.7%)		69 (56.1%)	25 (67.6%)	
≥ 70 years	31 (37.4%)	14 (58.3%)		54 (43.9%)	12 (32.4%)	
Median (IQR), years	66 (61–71)	70.5 (66–76.5)	0.033	68 (61–74)	68 (64–71)	0.754
ECOG PS			0.008			0.013
0–1	74 (89.2%)	16 (66.7%)		98 (79.7%)	22 (59.5%)	
2–3	9 (10.8%)	8 (33.3%)		25 (20.3%)	15 (40.5%)	
Smoking status			0.400			> 0.998
Never-smoker	1 (1.2%)	1 (4.2%)		2 (1.6%)	0 (0.0%)	
Current/former smoker	82 (98.8%)	23 (95.8%)		121 (98.4%)	37 (100.0%)	
Regimen			0.224			0.242
Etoposide and platinum	83 (100.0%)	23 (95.8%)		114 (92.7%)	32 (86.5%)	
Irinotecan and cisplatin	0 (0.0%)	1 (4.2%)		9 (7.3%)	5 (13.5%)	
Prophylactic cranial irradiation			0.132			0.006
Yes	49 (59.0%)	10 (41.7%)		50 (40.7%)	6 (16.2%)	
No	34 (41.0%)	14 (58.3%)		73 (59.4%)	31 (83.8%)	
Lactate dehydrogenase status (*n* = 72 in LD, *n* = 133 in ED)			0.339			0.474
Normal	31 (57.4%)	8 (44.4%)		34 (34.0%)	9 (27.3%)	
Elevated	23 (42.6%)	10 (55.6%)		66 (66.0%)	24 (72.7%)	
Median BMI (IQR), kg/m^2^	23.0 (20.8–25.2)	21.9 (20.1–23.8)	0.104	22.6 (20.7–24.5)	21.0 (19.9–22.5)	0.007

*LD* limited-stage disease, *ED* extensive-stage disease, *CXI* cachexia index, *IQR* interquartile range, *ECOG PS* Eastern Cooperative Oncology Group performance status, *BMI* body mass index

### Treatment response

Of 267 patients, 16 were not available for the radiological assessment of treatment response (Table 2). The ORRs in the low- and high-CXI groups were 79.2 and 95.2% (*p* = 0.026) for LD patients and 54.1 and 85.4% (*p* <  0.001) for ED patients, respectively. Only one LD patient and no ED patient achieved a CR in the low-CXI group. All 33 patients who achieved a CR completed their planned treatments. When 51 patients who discontinued treatment early (because of toxicity or patient decision) or for whom the radiological treatment response was not assessed were excluded from the analysis, the ORR remained lower in the low-CXI group compared to the high-CXI group regardless of stage (86.7% vs. 100.0%, respectively, *p* = 0.031, for LD patients; 66.7% vs. 86.2%, respectively, *p* = 0.022, for ED patients).

**Table 2:** Treatment responses

Treatment response	LD			ED		
	High-CXI group (*n* = 83)	Low-CXI group (*n* = 24)	*p*	High-CXI group (*n* = 123)	Low-CXI group (*n* = 37)	*p*
Complete response (CR)	30 (36.1%)	1 (4.2%)	0.004	2 (1.6%)	0 (0.0%)	< 0.001
Partial response (PR)	49 (59.0%)	18 (75.0%)		103 (83.7%)	20 (54.1%)	
Stable disease or progressive disease	1 (1.2%)	2 (8.3%)		15 (12.2%)	10 (27.0%)	
Not available	3 (3.6%)	3 (12.5%)		3 (2.4%)	7 (18.9%)	
Objective response rate (CR + PR)	79 (95.2%)	19 (79.2%)	0.026	105 (85.4%)	20 (54.1%)	< 0.001

*LD* limited-stage disease, *ED* extensive-stage disease, *CXI* cachexia index

### Treatment-related toxicity

Adverse treatment-related events are listed in Table 3. There were no significant differences in hematological toxicity between the low- and high-CXI groups. However, low-CXI patients received fewer cycles of chemotherapy (median 3 vs. 6 cycles, respectively, *p* <  0.001, for ED patients) and discontinued treatment early because of treatment-related toxicity more frequently (37.5% vs. 16.9%, respectively, *p* = 0.030, for LD patients; 27.0% vs. 11.4%, *p* = 0.019, for ED patients) compared to high-CXI patients. TRM tended to occur more frequently in the low-CXI group than in the high-CXI group for ED patients (10.8% vs. 2.4%, respectively, *p* = 0.051).

**Table 3:** Treatment-related toxicity and treatment compliance

Adverse event	LD			ED		
	High-CXI group (*n* = 83)	Low-CXI group (*n* = 24)	*p*	High-CXI group (*n* = 123)	Low-CXI group (*n* = 37)	*p*
Hematological toxicity ≥ grade 3						
Anemia	17 (20.5%)	7 (29.2%)	0.369	21 (17.1%)	9 (24.3%)	0.322
Neutropenia	78 (94.0%)	21 (87.5%)	0.375	108 (87.8%)	29 (78.4%)	0.152
Febrile neutropenia	16 (19.3%)	5 (20.8%)	> 0.998	17 (13.8%)	8 (21.6%)	0.252
Thrombocytopenia	21 (25.3%)	7 (29.2%)	0.704	27 (22.0%)	10 (27.0%)	0.521
Median treatment cycles (IQR)	6 (4–6)	5.5 (3–6)	0.118	6 (4–6)	3 (2–6)	< 0.001
Early discontinuation of treatment	14 (16.9%)	9 (37.5%)	0.030	14 (11.4%)	10 (27.0%)	0.019
Treatment-related mortality	4 (4.8%)	3 (12.5%)	0.186	3 (2.4%)	4 (10.8%)	0.051

*LD* limited-stage disease, *ED* extensive-stage disease, *CXI* cachexia index, *IQR* interquartile range

### Survival

**Fig. 1:**
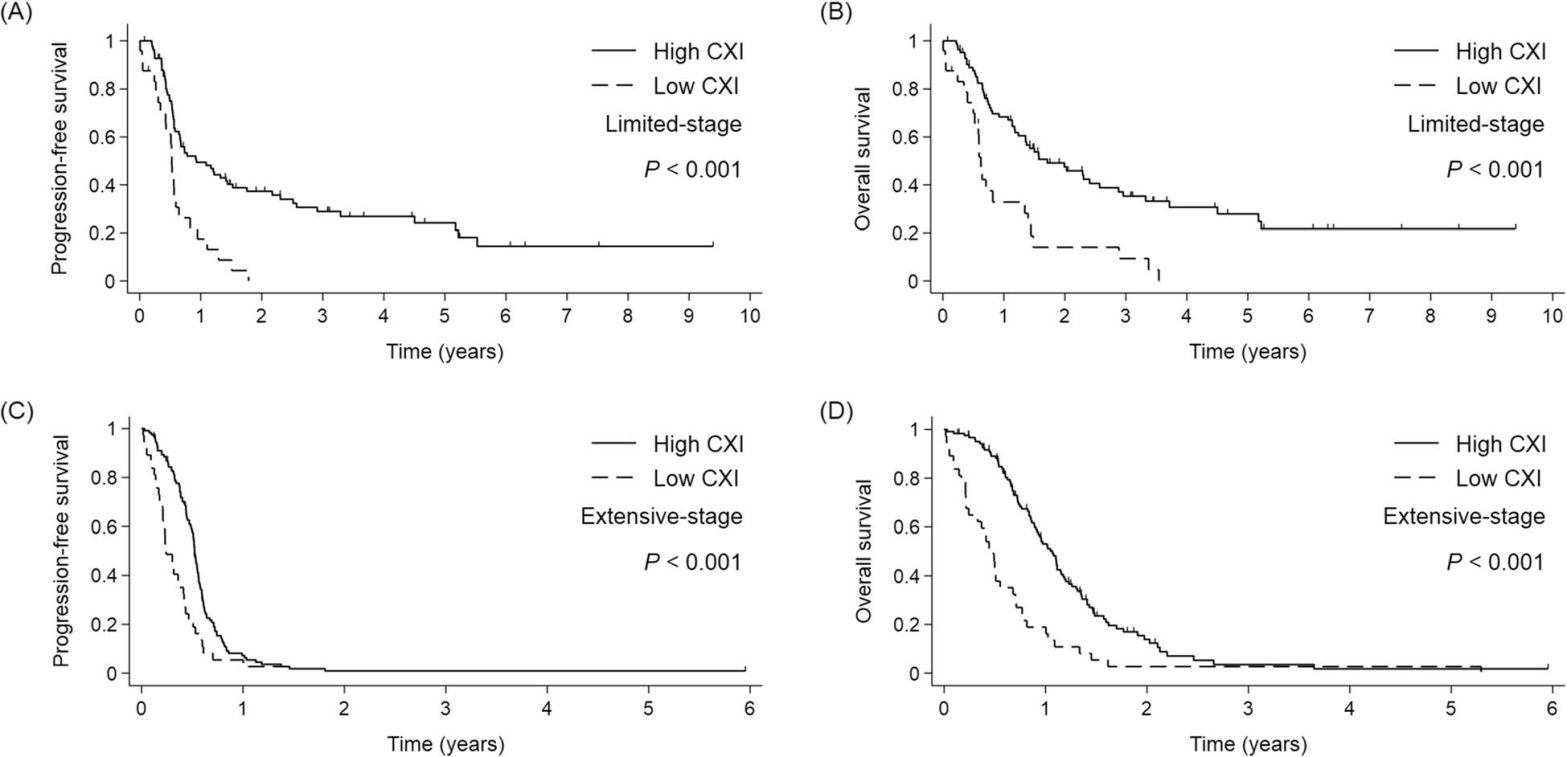
Kaplan-Meier curves for (**a**, **c**) progression-free survival and (**b**, **d**) overall survival according to the cachexia index (CXI) and tumor stage

The median follow-up duration was 41 months. Similar findings were obtained regardless of stage. In LD, patients with a low CXI had a shorter PFS (median 6.3 vs. 11.1 months, respectively, *p* <  0.001; Fig. 1a) and OS (median 7.5 vs. 20.6 months, respectively, *p* <  0.001; Fig. 1b) compared to those with a high CXI. In ED, patients with a low CXI also had a shorter PFS (median 2.9 vs. 6.3 months, respectively, *p* <  0.001; Fig. 1c) and OS (median 5.8 vs. 12.8 months, respectively, *p* <  0.001; Fig. 1d) compared to those with a high CXI.

On multivariate analysis, a low CXI was an independent poor prognostic factor for PFS [low CXI, hazard ratio (HR) 2.445, 95% CI 1.406–4.253, *p* = 0.002] and OS (low CXI, HR 2.393, 95% CI 1.372–4.174, *p* = 0.002) in LD patients. In ED patients, an ECOG PS of 2–3 and a low CXI were independent poor prognostic factors for PFS (low CXI, HR 1.764, 95% CI 1.195–2.604, *p* = 0.004) and OS (low CXI, HR 2.269, 95% CI 1.529–3.366, *p* <  0.001) (Table 4).

**Table 4:** Cox’s regression analysis for PFS and OS

Factor	PFS						OS					
	Univariate			Multivariate			Univariate			Multivariate		
	HR	95% CI	*p*	HR	95% CI	*p*	HR	95% CI	*p*	HR	95% CI	*p*
Limited-stage												
Age												
< 70 years	Ref.						Ref.					
≥ 70 years	1.147	0.742–1.774	0.537				1.343	0.848–2.125	0.208			
ECOG PS												
0–1	Ref.			Ref.			Ref.			Ref.		
2–3	1.821	1.047–3.167	0.034	1.329	0.730–2.421	0.352	2.108	1.192–3.730	0.010	1.579	0.855–2.915	0.144
Smoking												
Never-smoker	Ref.						Ref.					
Current/former smoker	1.697	0.235–12.279	0.600				1.587	0.219–11.506	0.648			
Regimen												
Etoposide and platinum	Ref.			Ref.			Ref.			Ref.		
Irinotecan and cisplatin	8.175	1.063–62.881	0.044	4.553	0.567–36.536	0.154	12.525	1.566–100.158	0.017	7.415	0.887–61.945	0.064
BMI												
Other (≥ 18.5 kg/m^2^)	Ref.						Ref.					
Underweight (< 18.5 kg/m^2^)	1.328	0.639–2.762	0.447				1.121	0.514–2.444	0.774			
CXI												
High	Ref.			Ref.			Ref.			Ref.		
Low	2.768	1.671–4.587	< 0.001	2.445	1.406–4.253	0.002	2.830	1.698–4.717	< 0.001	2.393	1.372–4.174	0.002
Extensive-stage												
Age												
< 70 years	Ref.						Ref.					
≥ 70 years	1.088	0.786–1.506	0.612				1.297	0.906–1.855	0.155			
ECOG PS												
0–1	Ref.			Ref.			Ref.			Ref.		
2–3	2.083	1.435–3.023	< 0.001	1.819	1.236–2.679	0.002	3.152	2.121–4.684	< 0.001	2.768	1.847–4.149	< 0.001
Smoking												
Never-smoker	Ref.						Ref.					
Current/former smoker	1.071	0.264–4.344	0.924				0.989	0.244–4.016	0.988			
Regimen												
Etoposide and platinum	Ref.						Ref.					
Irinotecan and cisplatin	0.819	0.471–1.426	0.481				0.779	0.434–1.396	0.401			
BMI												
Other (≥ 18.5 kg/m^2^)	Ref.						Ref.					
Underweight (< 18.5 kg/m^2^)	1.243	0.775–1.994	0.367				1.052	0.631–1.754	0.847			
CXI												
High	Ref.			Ref.			Ref.			Ref.		
Low	2.039	1.401–2.967	< 0.001	1.764	1.195–2.604	0.004	2.644	1.796–3.894	< 0.001	2.269	1.529–3.366	< 0.001

*PFS* progression-free survival, *OS* overall survival, *HR* hazard ratio, *CI* confidence interval, *ECOG PS* Eastern Cooperative Oncology Group performance status, *BMI* body mass index, *CXI* cachexia index

## Discussion

This is the first study to report that a low CXI is closely related to poor clinical outcomes in SCLC patients. The CR rate and ORR differed greatly between the low- and high-CXI groups. Although the ECOG PS, which is an important prognostic factor, was not balanced between the two groups, PFS and OS were much poorer in the low-CXI group even after adjusting for the PS. Although the role for the CXI has not yet been examined in SCLC patients, each CXI factor has been suggested to be prognostic in several studies. Sarcopenic SCLC patients experienced poorer survival than non-sarcopenic patients [18]. When models that included both sarcopenia and levels of inflammatory markers were used, the clinical significance of sarcopenia was emphasized [19, 29]. Hypoalbuminemia and other indices reflecting low serum albumin levels were associated with reduced survival, increased treatment-related toxicity, and a low treatment response rate [21, 22, 30–32]. A high NLR was related to poor PS, a high probability of recurrence, and reduced survival [33–35]. The results of the present study and previous studies thus suggest that the CXI is significantly prognostic in SCLC patients.

Cancer cachexia must be carefully assessed. Percentage weight loss alone is of limited utility in the era of obesity [36, 37]. Sarcopenia has been used to diagnose and stage cancer cachexia [11]. Cross-sectional CT optimally assesses muscle mass [11]. Inflammatory cytokines produced by tumor cells [tumor necrosis factor (TNF)-ɑ, interleukin (IL)-6, and IL-8] contribute to muscle wasting by inducing oxidative stress in skeletal muscles and activating muscle degradation pathways [38–40]. TNF-ɑ inhibited albumin expression in a murine model of cachexia even before the onset of weight loss [41]. Ideal biomarkers of cancer cachexia must therefore reflect these various processes. Several studies have explored the clinical utilities of composite biomarkers or scoring systems for cachexia in SCLC patients [20–22, 32]. However, unlike the CXI, the biomarkers did not consider either sarcopenia or systemic inflammation. Although the CXI is more complex than other biomarkers, the factors can be measured non-invasively via routine baseline imaging and laboratory tests. The CXI may serve as an ideal biomarker of cachexia in SCLC patients.

The low-CXI group very frequently discontinued treatment early because of treatment-related toxicity and suffered a higher rate of TRM. A recent study reported that malnourished patients exhibited increased rates of toxicity of grade ≥ 3 and were more likely to be hospitalized in phase I and II oncology clinical trials [42]. Several studies found that sarcopenia is a significant predictor of dose-limiting toxicity in patients with various malignancies [43–46]. Chemotherapy doses are generally based on body surface area. Therefore, among patients of the same height and weight, sarcopenic patients receive relatively higher doses than do non-sarcopenic patients because drug metabolism occurs predominantly in lean body tissue [37]. Given that no patient who did not complete planned treatment achieved a CR, the treatment intolerance observed in the low-CXI group may be associated with a poor response to chemotherapy.

Our work had several limitations. First, the data were collected retrospectively and thus associated with a risk of selection bias. We also lacked detailed toxicity profiles. Second, the utility of the CXI in female patients could not be assessed in this study. Third, the method used to measure the SMI in the present study was different from the methods used in previous studies, and calculation of the CXI using the SMI of pectoralis muscle was not validated in SCLC, rendering comparisons difficult. Given the low sensitivity of the CXI cutoffs in the present study, validation of our methodological approach is essential. In the original study, Jafri et al. calculated the CXI and SMI using the skeletal muscle area as determined on abdominal CT in non-small cell lung cancer patients [23]. In our institution, abdominal CT is not a routine staging work-up for SCLC, and chest CT does not generally include the L3 level, which is the standard level use for calculation of the SMI. Instead, we examined the pectoralis muscle at the T4 level to calculate the SMI. In previous studies, SMIs calculated using muscles at other vertebral levels, or the pectoralis muscle, were strongly correlated with the L3-SMI [47–49]. In addition, previous studies used the median to determine the CXI cutoff [23, 24]. The time-dependent ROC analysis employed in the present study is an efficient means of measuring the performance of a biomarker at a certain time point with survival data compared with other methods, although censoring is still problematic [26]. Further large prospective studies are needed to validate our methodological approach and confirm the clinical applicability of the CXI in SCLC.

In conclusion, we found that cachectic patients with low CXIs more frequently discontinued treatment early and exhibited poor prognosis in SCLC. Intensive supportive care, including aggressive nutritional support and dose adjustment, may improve the treatment outcomes of SCLC patients with a low CXI receiving chemotherapy or chemoradiotherapy. We anticipate that the CXI may be adopted as an ideal biomarker for research on cancer cachexia and that its measurement will be integrated into routine practice for the diagnostic work-up for SCLC after validation through further prospective studies.

## Supplementary Information

**Additional file 1.** Time-dependent receiver operating characteristic (ROC) curve of the cachexia index (CXI) for prediction of (A) 18-month overall survival in limited-stage disease (LD) and (B) 10-month overall survival in extensive-stage disease (ED). Circles indicate CXIs of (A) 5.82 in LD and (B) 3.83 in ED. These cutoffs were determined by maximizing the Youden index.

## Data Availability

The datasets used and/or analyzed during the current study are available from the corresponding author on reasonable request.

## Abbreviations

AUC: Area under the curve
BMI: Body mass index
CI: Confidence interval
CR: Complete response
CT: Computed tomography
CXI: Cachexia index
ECOG PS: Eastern Cooperative Oncology Group performance status
ED: Extensive-stage disease
HR: Hazard ratio
IL: Interleukin
LD: Limited-stage disease
NLR: Neutrophil-to-lymphocyte ratio
ORR: Objective response rate
OS: Overall survival
PFS: Progression-free survival
PR: Partial response
RECIST: Response Evaluation Criteria in Solid Tumors
ROC: Receiver operating characteristic
SCLC: Small-cell lung cancer
SMI: Skeletal muscle index
TNF: Tumor necrosis factor
TRM: Treatment-related mortality
