# Supplementary material for: Clinical significance of the cachexia index in patients with small cell lung cancer
Source: BMC Cancer. 2021 May 17;21:563. doi: 10.1186/s12885-021-08300-x (PMC8130111; doi:10.1186/s12885-021-08300-x)
Supplement: Supplementary file 1 — Additional file 1. Time-dependent receiver operating characteristic (ROC) curve of the cachexia index (CXI) for prediction of (A) 18-month overall survival in limited-stage disease (LD) and (B) 10-month overall survival in extensive-stage disease (ED). Circles indicate CXIs of (A) 5.82 in LD and (B) 3.83 in ED. These cutoffs were determined by maximizing the Youden index. [file 12885_2021_8300_MOESM1_ESM.docx]

**Supplementary file information**

**File name:**


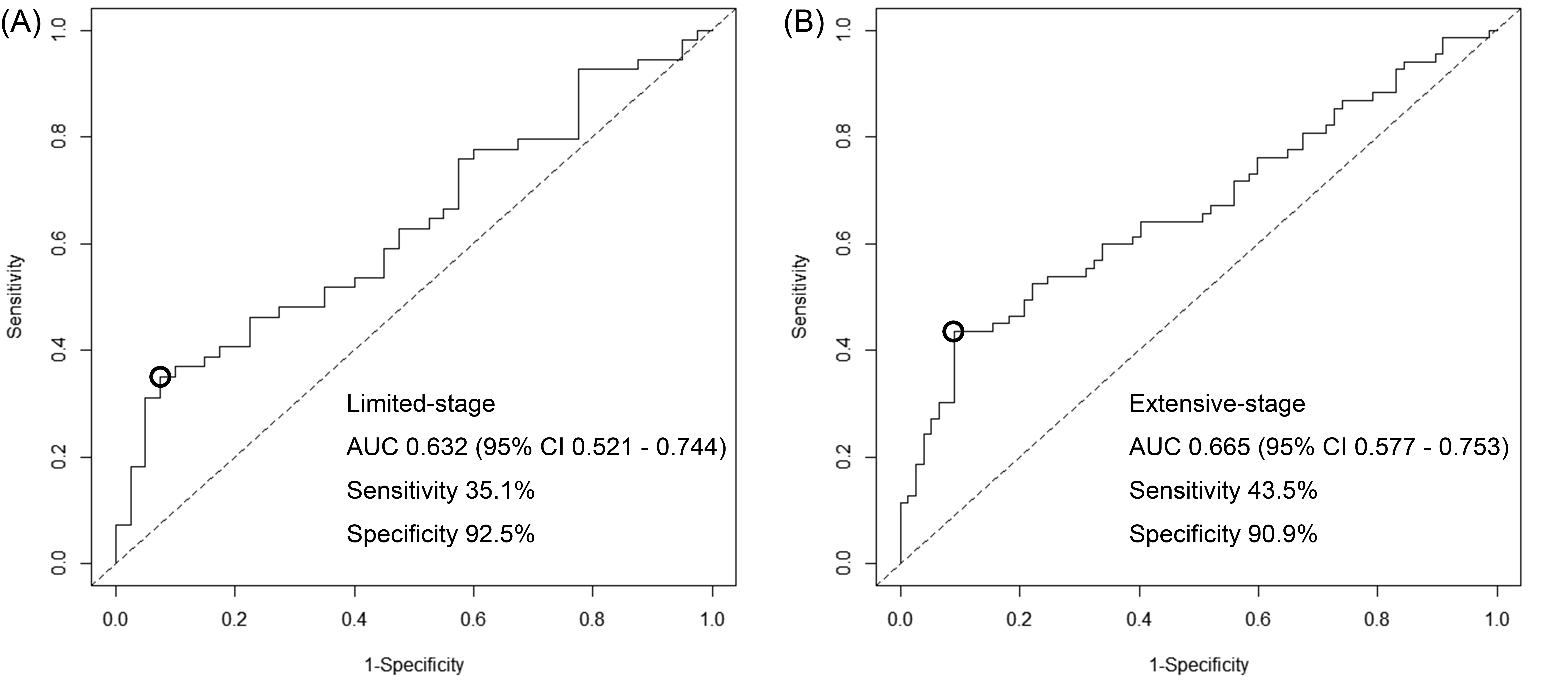


supplementary figure 1.clinical significance of the cachexia index in patients with small cell lung cancer by Se-Il Go, Mi Jung Park, Gyeong-Won Lee

**File format:**

.docx

**Title of data:**

Time-dependent receiver operating characteristic (ROC) curve of the cachexia index (CXI) for prediction of (A) 18-month overall survival in limited-stage disease (LD) and (B) 10-month overall survival in extensive-stage disease (ED).

**Description of data:**

Circles indicate CXIs of (A) 5.82 in LD and (B) 3.83 in ED. These cutoffs were determined by maximizing the Youden index.
